# *Campylobacter jejuni* Response When Inoculated in Bovine In Vitro Fecal Microbial Consortia Incubations in the Presence of Metabolic Inhibitors

**DOI:** 10.3390/pathogens12121391

**Published:** 2023-11-26

**Authors:** Dana K. Dittoe, Robin C. Anderson, Nathan A. Krueger, Roger B. Harvey, Toni L. Poole, Tawni L. Crippen, Todd R. Callaway, Steven C. Ricke

**Affiliations:** 1 Department of Animal Science, University of Wyoming, Laramie, WY 82071, USA; ddittoe@uwyo.edu; 2 United States Department of Agriculture, Agricultural Research Service, Southern Plains Agricultural Research Center, College Station, TX 77845, USA; robin.anderson@usda.gov (R.C.A.); roger.harvey@usda.gov (R.B.H.); toni.poole@usda.gov (T.L.P.); tc.crippen@usda.gov (T.L.C.); 3 Agricultural Sciences, Blinn College, Bryan, TX 77833, USA; nathan.krueger@blinn.edu; 4 Ruminant Nutrition, Ruminant Microbiology, and Preharvest Food Safety, Department of Animal and Dairy Science, University of Georgia, Athens, GA 30602, USA; todd.callaway@uga.edu; 5 Meat Science and Animal Biologics Discovery Program, Department of Animal and Dairy Sciences, University of Wisconsin, Madison, WI 53706, USA

**Keywords:** *Campylobacter jejuni*, rumen incubations, fecal suspensions, anti-methane compounds

## Abstract

Infection with the foodborne pathogen *Campylobacter* is the leading bacterial cause of human foodborne illness in the United States. The objectives of this experiment were to test the hypothesis that mixed microbial populations from the bovine rumen may be better at excluding *Campylobacter* than populations from freshly voided feces and to explore potential reasons as to why the rumen may be a less favorable environment for *Campylobacter* than feces. In an initial experiment, *C. jejuni* cultures inoculated without or with freshly collected bovine rumen fluid, bovine feces or their combination were cultured micro-aerobically for 48 h. Results revealed that *C. jejuni* grew at similar growth rates during the first 6 h of incubation regardless of whether inoculated with the rumen or fecal contents, with rates ranging from 0.178 to 0.222 h^−1^. However, *C. jejuni* counts (log_10_ colony-forming units/mL) at the end of the 48 h incubation were lowest in cultures inoculated with rumen fluid (5.73 log_10_ CFUs/mL), intermediate in cultures inoculated with feces or both feces and rumen fluid (7.16 and 6.36 log_10_ CFUs/mL) and highest in pure culture controls that had not been inoculated with the rumen or fecal contents (8.32 log_10_ CFUs/mL). In follow-up experiments intended to examine the potential effects of hydrogen and hydrogen-consuming methanogens on *C. jejuni*, freshly collected bovine feces, suspended in anaerobic buffer, were incubated anaerobically under either a 100% carbon dioxide or 50:50 carbon dioxide/hydrogen gas mix. While *C. jejuni* viability decreased <1 log_10_ CFUs/mL during incubation of the fecal suspensions, this did not differ whether under low or high hydrogen accumulations or whether the suspensions were treated without or with the mechanistically distinct methanogen inhibitors, 5 mM nitrate, 0.05 mM 2-bromosulfonate or 0.001 mM monensin. These results suggest that little if any competition between *C. jejuni* and hydrogen-consuming methanogens exists in the bovine intestine based on fecal incubations.

## 1. Introduction

*Campylobacter* has been isolated from most food-producing animals with a particularly high prevalence in swine [1,2] and poultry [3,4,5,6] and ranging from low levels to greater than 89% prevalence in ruminants [7]. *Campylobacter* spp. can colonize the gastrointestinal tracts of food-producing animals as well as wild and feral animals [2,5]. *Campylobacter jejuni* is the predominant species associated with poultry and cattle [7]. *Campylobacter coli* is recognized as the primary species in swine [7]; however, considerable numbers of pigs can be colonized with *C. jejuni* [8,9].

*Campylobacter* originating from food animals continues to be a major public health concern and a leading cause of human gastrointestinal diseases both in the United States as well as worldwide [6,10,11]. In addition, in some human cases, *Campylobacter* has been associated with post-infection involving immune-mediated neuropathies known as Guillian Barré Syndrome [6]. *Campylobacter* is also recognized as a reservoir of genes that encode for resistance to antibiotics that are important for the clinical treatment of human disease [12]. With the emergence of molecular methodologies such as whole-genome sequencing and more advanced applications of polymerase chain assays for rapid detection, there have been an increasing number of species and subspecies of *Campylobacter* identified in a wide range of sources [13]. However, *C. jejuni* remains the cause of most of the human illness cases, followed by *C. coli* [11].

*Campylobacter* associated with ruminants represents a potential public health concern along with poultry and swine [2,5]. Dairy sources have been reported to be one of the highest causes of campylobacteriosis in humans, and *C. jejuni* isolates bearing a close genetic relationship with human strains have been detected in dairy cattle fecal material as well as environmental samples and exhibit increased cases in rural communities with high densities of dairy cattle [14,15]. Further concerns have been raised when some *Campylobacter* spp. including *C. jejuni* isolates from dairy farms exhibit antibiotic resistance and/or carry the lipooligosaccharide classes potentially responsible for triggering Guillian Barré Syndrome [16,17]. Beef cattle farms, feedlots, and cow–calf operations have also been proven to be sources of *Campylobacter* that display antibiotic resistance [18,19,20,21]. This may also be a public health concern as antibiotic-resistant *Campylobacter* spp. have been isolated from beef products such as beef livers [22,23,24]. In cattle, *Campylobacter* is more likely to colonize the lower gastrointestinal tract and has exhibited only limited ability to survive in rumen in vitro incubations [25,26]. This would suggest that fecal sources may be more important as a vector for *Campylobacter* in cattle than the rumen and upper gastrointestinal tract populations. Thus, the objectives of this experiment were to test the hypothesis that mixed microbial populations from the bovine rumen may be better at excluding *Campylobacter* than populations from freshly voided feces and to explore potential reasons as to why the rumen may be a less favorable environment for *Campylobacter* than feces.

## 2. Materials and Methods

### 2.1. Microbial Sources

A poultry field isolate of *Campylobacter jejuni* was used in these studies [27]. *Campylobacter jejuni* used as an inoculum was grown in 18 × 150 mm crimp-top culture tubes containing nonantibiotic supplemented Bolton broth (Oxoid Ltd., Basingstoke, UK) as described by Anderson et al. [28] that was flushed with an microaerobic gas phase (10% CO_2_, 5% O_2_, 85% N_2_) and incubated 24 h at 42 °C to yield approximately 10^8^ colony-forming units (CFUs)/mL as the final concentration. Collections of bovine ruminal and fecal micro-organisms were conducted on the morning of each experiment (10:00 AM) from a ruminally cannulated Jersey cow that was not lactating and grazing on coastal bermudagrass pasture. Rumen contents withdrawn from the cannula were strained through a nylon paint strainer into an insulated container until full and capped immediately to minimize oxygen exposure. Feces collected via rectal palpation were placed into a Nasco Whirl-Pak^®^ (Madison, WI, USA) and immediately closed. Transport of rumen fluid and feces to the laboratory occurred within 30 min of collection. Measurements of pH of undiluted rumen fluid and freshly diluted fecal suspensions (8% *w*/*v* diluted in water) using a pH meter ranged between 6.34 to 6.53. *Campylobacter* status of the freshly collected rumen and fecal samples, was determined by plating 1 mL or 1 g portions of each freshly collected sample combined with 1 mL 0.1 M phosphate buffer (pH 6.5) on Campy Cefex agar as described previously [29]. The presence or absence of *Campylobacter* colonies was confirmed after 48 h microaerobic incubation. Husbandry procedures for animal care were approved by the Southern Plains Agricultural Research Center’s Animal Care and Use Committee.

### 2.2. Comparison of C. jejuni in Fecal versus Rumen Mixed Microbial Populations

A *Campylobacter jejuni* culture grown for 24 h was added (0.35 mL) to a 350 mL batch of freshly prepared Bolton broth amended to contain glucose, cellobiose and xylose (Sigma-Aldrich, St. Louis, MO, USA), each at 0.2% *w*/*v*. The carbohydrates were added to the Bolton broth to serve as substrates for rumen micro-organisms. Ten-milliliter volumes of the *C. jejuni*-inoculated, sugar-amended Bolton broth were then distributed under a continuous flow of microaerobic gas mix (10 % CO_2_, 5 % O_2_, 85 % N_2_) to presterilized 18 × 150 mm crimp-top culture tubes. Triplicate sets of the culture tubes were subsequently inoculated individually (0.2 mL) or jointly (0.1 mL each) with suspensions of the freshly collected gastrointestinal tract populations, previously serially 10-fold-diluted in anaerobic dilution solution [30] to 1:10,000, to compare effects of the bovine rumen, bovine fecal micro-organisms or their combination. The freshly collected bovine rumen and fecal samples were each diluted to deplete endogenous substrate and to dilute to extinction the potential effect of wildtype *Campylobacter* that may have been present in the samples. Once all additions to the culture tubes were added, the tubes were closed with rubber stoppers, crimped, and incubated at 39 °C for 48 h under the 10% CO_2_, 5% O_2_, 85% N_2_ headspace gas phase. During incubation, samples (1 mL) were collected from each tube at 0, 6, 24 and 48 h for colorimetric measurement of ammonia [31] and enumeration of *Campylobacter* via plating of serial 10-fold dilutions (in 0.1 M phosphate buffer, pH 6.5) to Campy Cefex agar. Colonies exhibiting typical *Campylobacter* morphology on the Campy Cefex agar were counted after 48 h of incubation at 42 °C. Representative colonies picked at random during mixed culture studies were confirmed as *Campylobacter* based on the amplification and detection of the *ceuE* gene [32]. Additionally, the number of total anaerobes was quantified in the original diluted fecal and ruminal fluid samples used as treatments as well as in samples collected from each culture tube at the end of the 48 h incubation period via plating of serial 10-fold dilutions to anaerobic Brucella Blood Agar (Anaerobe Systems, Morgan Hill, CA, USA) as performed earlier [28]. Dilution and plating of the fluid samples for enumeration of anaerobes and subsequent incubation (39 °C for 48 h) of inoculated Brucella agar were carried out in a Bactron IV Anaerobic Environmental Chamber (Sheldon Manufacturing Inc., Cornelius, OR, USA) under an 85% N_2_, 15% CO_2_ and 5% H_2_ atmosphere.

### 2.3. Impact of Different Gas Atmospheres and Anti-Methanogenic Treatments on C. jejuni Survivability and Select Incubation Characteristics in Mixed Rumen and Fecal Populations

For experimental incubations with the anti-methanogenic treatments 2-bromosulfonate and nitrate, 80 g of freshly collected feces were suspended in 500 mL anaerobic dilution solution supplemented with 35 g Bacto^TM^ casamino acids (Becton, Dickinson and Co., Sparks, MD, USA) to achieve a 14% *w*/*v* fecal suspension containing 7% casamino acids. The suspension was mixed vigorously via 15 min of high-speed stirring on a stir plate while continuously under 100% carbon dioxide and then separated into two equal volume batches, with one batch being inoculated with 0.3 mL of a 1:10 dilution of *C. jejuni* grown overnight but not the other batch. After mixing, 10 mL volumes of each fecal suspension were transferred to 18 × 150 mm glass tubes preloaded without or with 0.2 mL of stock concentrations of 24 mM 2-bromosulfonate, 250 mM nitrate (each in water) or both to achieve initial concentrations at the start of incubation of 0.001, 0.05 or their respective combinations. Depending on the experimental design, all additions were made to the tubes while under a continuous flow of either 100% carbon dioxide or a mixture of 50:50 hydrogen/carbon dioxide with each tube subsequently being closed by a rubber stopper and immediately sealed after introducing all additions. Experimental incubations testing the effects of 0.05 mM 2-bromosulfonate, 0.001 mM sodium monensin (both from Sigma-Aldrich) or their combination were conducted similarly except with a fecal suspension inoculated with 10% *w*/*v* of freshly collected feces. Monensin, being poorly soluble in water, was added at 0.1 mL from a 0.1 mM stock solution prepared in ethanol. For consistency, 0.1 mL ethanol (Sigma-Aldrich) was added to untreated controls and 2-bromosulfonate-treated incubations. The fecal suspension contained 7% casamino acids as above, but for these incubations only carbon dioxide was used (100% was the only gas phase tested). For all incubations, tubes were maintained upright with no agitation at 39 °C for 24 h. Samples of 1.5 mL fluid were removed from each tube with separate 1 cc syringes at 0, 6 and 24 h for colony enumeration of *Campylobacter* on Campy Cefex agar via serial 10-fold dilution of 1 mL aliquots from each sample. Portions of the remaining fluid samples from 0 and 24 h collections were used for colorimetric determination of nitrate concentrations [33]. At the end of the 24 h incubation, 1 mL headspace was collected from each tube and analyzed via gas chromatography on a Gow-Mac gas chromatograph as described previously [34]. Gas volumes in each tube were measured via volume displacement using a 20 mL gas-tight glass syringe and gas concentrations were calculated using the ideal gas law.

### 2.4. Statistical Analysis

Statistical analyses of *Campylobacter* counts as log_10_ transformations were conducted at each sampling time for impact of microbial populations or their mixtures using a general analysis of variance. Ammonia concentrations, pH and log_10_ transformations of total culturable anaerobes measured at the end of the 48 h incubations were likewise analyzed for effects on microbial populations or their mixtures. In the second experiment, comparisons of the main effects of the gas phase, anti-methanogen treatment, log_10_ concentrations of *C. jejuni* or their potential interactions were likewise analyzed via general analysis of variance. Total gas generated, hydrogen and methane final concentrations and the net change in *Campylobacter jejuni* populations were determined in *C. jejuni* inoculated cultures. Main effect comparisons of *C. jejuni* inoculation, treatment and their potential interaction were determined in cultures that had been either inoculated or not inoculated with *C. jejuni* for total gas produced, final hydrogen, and methane concentrations. To avoid confounding effects of the supplied gas phases, separate comparisons were conducted on incubations initiated under 100% carbon dioxide or the 50:50 hydrogen/carbon dioxide mix. Results from the third experiment testing the effects of 2-bromosulfonate, monensin or their combination were analyzed similarly except, due to the presence of wildtype *Campylobacter*, the comparisons of the main effects of *C. jejuni* inoculation and relevant interactions were omitted. When detected, physiologically significant interactions between the gas phase and anti-methanogenic treatments or *Campylobacter* and anti-methanogenic treatments were noted. When the main effects or interactions were significant, means were separated based on an LSD multiple comparison of means. All analyses were conducted using Statistix version10 Analytical Software (Tallahassee, FL, USA).

## 3. Results

### 3.1. Comparison of C. jejuni Survival in Mixed Rumen and Bovine Fecal In Vitro Microbial Cultures

**Figure 1:**
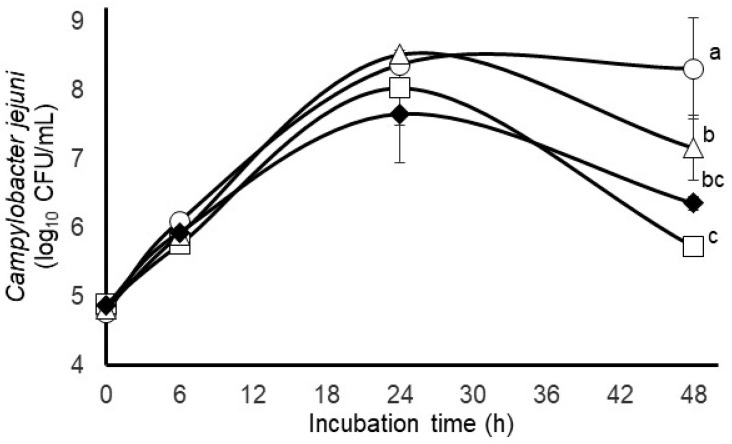
Comparison of growth/survival characteristics of *Campylobacter jejuni* grown in pure culture (circles) or with mixed populations of bovine rumen micro-organisms (squares), bovine fecal micro-organisms (triangles) or their combination (closed diamonds). Values at each timepoint are least-squares means ± standard deviations from cultures incubated in triplicate. Means with different letter affiliations differ at *p* < 0.05 based on an LSD multiple comparison of means.

No wildtype background *Campylobacter* were detected in bovine or fecal samples prior to use in these initial set of experiments. The growth curves for *C. jejuni* in eitherpure culture or experimentally inoculated into the bovine rumen or fecal micro-organisms mixed populations are presented in Figure 1. In support of our hypothesis that rumen micro-organisms may be more antagonistic to *C. jejuni* than fecal micro-organisms, we observed that the *C. jejuni* concentrations measured at the end of the 48 h incubation period were more than 2.5 log_10_ CFUs/mL lower (*p* < 0.05) in the mixed culture with rumen micro-organisms than in the pure culture and 1.4 log_10_ units lower in the mixed culture with fecal micro-organisms (Figure 1). The antagonistic effect of the rumen micro-organisms against *C. jejuni* appeared to be restricted to the later incubation period; however, *C. jejuni* counts did not differ between the pure or mixed cultures (*p* > 0.05) when measured at 0, 6 or 24 h of incubation (Figure 1). Similarly, the mean specific growth rates of *C. jejuni* determined over the first 6 h of incubation did not differ (*p* > 0.05) between the mixed or pure cultures, further indicating a near-unimpeded growth of *C. jejuni* during the early incubation periods (Table 1). The concentrations of rumen and fecal anaerobes, initially at 4.0 and 3.0 log_10_ CFUs/mL at the start of the incubations, did not differ (*p* > 0.05) between the mixed cultures after 48 h incubation (Table 1). The pH of the mixed population incubations of rumen or fecal micro-organisms or their combinations cultured with *C. jejuni*, initially at pH 6.50, were lower (*p* < 0.05) after 48 h incubation than the pure cultures of *C. jejuni* incubated likewise (Table 1). Based on the 3 to 4 log-fold increase in total anaerobes and the resulting decrease in pH during the incubations, likely due to the greater fermentative activity of the rumen and fecal anaerobes, it is reasonable to suspect that given sufficient time, the anaerobes were able to actively compete against the inoculated *C. jejuni*. However, net accumulations of ammonia did not differ (*p* > 0.05) between the pure *C. jejuni* cultures or any of the mixed microbial populations cultured with *C. jejuni*, thus revealing no obvious disruption in ammonia production or consumption.

### 3.2. Impact of Different Gas Atmospheres and Anti-Methanogenic Treatments on C. jejuni Survivability and Select Incubation Characteristics in Mixed Fecal Populations

**Figure 2:**
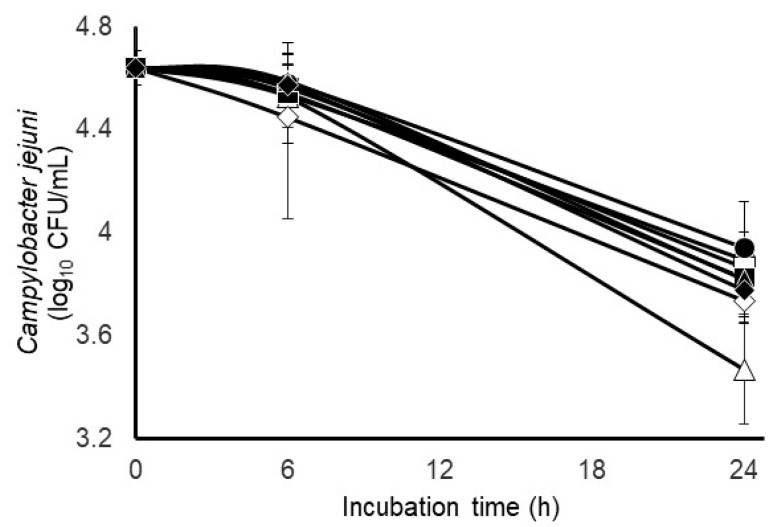
Comparison of survival characteristics of experimentally inoculated *Campylobacter jejuni* during culture with mixed populations of bovine fecal micro-organisms initiated with 100% carbon dioxide (open symbols) or a 50:50 hydrogen/carbon dioxide gas phase and treated without (circles) or with 0.05 mM 2-bromosulfonate (squares), 5 mM sodium nitrate (triangles) or their combination (diamonds). Values at each timepoint are least-squares means ± standard deviation from cultures incubated in triplicate.

The quantitative results from the bacteriological culture of the bovine feces collected for this particular experiment revealed the presence of 1.1 × 10^5^ CFUs of wildtype *Campylobacter*/g of feces; consequently, only mixed fecal populations that had been inoculated with *C. jejuni* were analyzed and no attempt was made to differentiate the inoculated *C. jejuni* strain from the wildtype. The survival characteristics of *C. jejuni* during incubation with mixed populations of bovine fecal microbial populations under two initially different gas phases (carbon dioxide or 50:50 hydrogen/carbon dioxide) and without or with two anti-methanogenic treatments that would be considered mechanistically distinct, as well as their combination, are provided in Figure 2. No main effect of the gas phase was observed on the net change in *Campylobacter* concentrations after 24 h incubation of the mixed fecal populations. A main effect of gas phase was observed on total gas production, hydrogen and methane accumulation but not metabolized nitrate (Table 2). The amount of total gas volume and methane produced was slight in the fecal incubations. A main effect of anti-methanogen treatment was not observed (*p* > 0.05) on methane production, but a tendency for a treatment effect was observed on the net change in *Campylobacter* concentrations, with survivability appearing to be lowest after 24 h (but not 6 h) incubation of the mixed fecal populations treated with 5 mM sodium nitrate (Table 2). Less total gas was also produced by the fecal incubations treated individually with 5 mM nitrate or combined with 0.05 mM 2-bromosulfonate. The main effects of initial gas phase, 100% carbon dioxide or a 50:50 carbon dioxide/hydrogen mix, along with mechanistically different anti-methanogenic compounds bromosulfonate and monensin, administered alone or together to mixed fecal micro-organisms are presented in Table 3. Gas phase or anti-methanogenic compound did not statistically impact (*p* > 0.05) *C. jejuni* populations after 6 h, or total gas production, hydrogen. methane, and ammonia accumulation after 24 h.

## 4. Discussion

*Campylobacter* spp. have been isolated from a wide range of animals, farm, and urban environments as well as meat-processing plants [5]. *Campylobacter* are especially known for their high prevalence in cattle, swine, and poultry, leading to concerns over food safety when these animals are slaughtered, with the majority of disease outbreaks occurring from raw or undercooked meat products [5]. Poultry products have been considered one of the primary sources of campylobacteriosis, which is consistent with the ability of *Campylobacter* to readily colonize gastrointestinal tract populations and interact with the indigenous microbiota [5,35]. However, cattle-based meat products can also serve as a source of *Campylobacter* and can be detected in ruminants under a range of management conditions [2,36]. However, the ecology and sites of colonization in the gastrointestinal tract are not well known.

Although no indigenous *Campylobacter* were detected in rumen contents, inoculated *C. jejuni* could survive in rumen contents (Figure 1). However, based on the results presented in Figure 1, it appears that rumen micro-organisms are more antagonistic to *C. jejuni* than fecal micro-organisms, as *C. jejuni* levels at the end of the 48 h incubation period were 1.4 log_10_ CFUs/mL less in the presence of rumen micro-organisms than when incubated with fecal micro-organisms. This reduction was evident only at the end of the fermentation as no differences in mixed cultures of *Campylobacter* versus pure cultures were detected at earlier timepoints. Since the total anaerobic microbial populations did not change over time, this decrease may be due to a combination of specific nutrient limitations being reached for *Campylobacter* after 24 h in mixed cultures, but with sufficient nutrients left to sustain the general microbial rumen and fecal communities and/or the buildup of fermentative products that are antagonistic to *Campylobacter*. Decreases in pH at 48 h (Table 1) suggest that an accumulation of fermentation products over time that are antagonistic to *Campylobacter* is possible as organic acids have been identified as one of the mechanisms associated with anti-*Campylobacter* probiotic cultures [37].

It is less clear whether nutrient limitation was a factor as detectable increases in ammonia did not differ (Table 1) between the pure *C. jejuni* cultures or any of the mixed microbial populations cultured with *C. jejuni*. Presumably, amino acid fermentation would lead to increases in ammonia for pure cultures of *C. jejuni* since Mueller–Hinton agar contains 300 g dehydrated beef extract and 17.5 g casein hydrolysate per liter. However, it is conceivable that either these sources of amino acid were not optimal for *C. jejuni* to use these pathways or other substrates served as primary sources of carbon and energy. This could also be reflective of *C. jejuni’s* limited metabolic capabilities. Rath et al. [38] examined the metabolomic profiles of intestinal contents of pigs inoculated with either *C. jejuni* or *C. coli* and reported that *C. coli* could use a wide range of substrates including short-chain fatty acids, fucose, as well as serine and asparagine, while *C. jejuni* could only use serine. It would be of interest in future mixed fecal culture incubation studies to compare ammonia production from various *Campylobacter* spp., particularly *C. coli* versus *C. jejuni*.

In the current study, *in vitro* rumen and fecal incubations were used in the presence of methanogen inhibitors and nitrate to determine if there is a relationship between *C. jejuni* and the methanogenic population as a function of hydrogen. The survival characteristics of *C. jejuni* were not detectably altered (Figure 2) during incubation with mixed populations of bovine fecal micro-organisms under two initially different gas phases (carbon dioxide or 50:50 hydrogen/carbon dioxide) and without or with two mechanistically different anti-methanogenic treatments, or their combination. Unlike that observed with mixed rumen populations [26], a main effect of the gas phase was not observed on the net change in *Campylobacter* concentrations after 24 h incubation of the mixed fecal populations. However, a main effect of the gas phase was observed on total gas production, hydrogen and methane accumulation in the fecal incubations (Table 2). Compared to hydrogen accumulation, the relative levels of total gas and methane were modest, but this could have been associated with minimal methanogen activity occurring in fecal contents as opposed to what would typically be seen in rumen incubations. This is consistent with the lack of a detectable impact of anti-methanogen compounds on methane production. However, less total gas was also produced by the fecal incubations treated individually with 5 mM nitrate or combined with 0.05 mM 2-bromosulfonate, while only the combined compounds reduced hydrogen significantly. The anti-methanogenic compounds did apparently decrease *Campylobacter*’s survivability, with the lowest numerical levels in the fecal incubations treated with 5 mM sodium nitrate (Table 2).

It is not clear whether *Campylobacter* can become established in the mature rumen, but there is evidence that it can survive under certain circumstances such as in the presence of protozoa [35]. *Campylobacter* have also been shown to exhibit a trend to increase in the rumens of beef cattle undergoing preslaughter fasting [39]. The factors that influence the presence of *Campylobacter* in the rumen are unclear but 16S rDNA microbiome taxonomic data from previous poultry *in vitro* cecal incubation studies detected a potential inverse relationship between methanogens and *Campylobacter* [40]. In addition, metagenomics profiling of poultry cecal microbiota indicated that *Campylobacter* possessed uptake hydrogenases and was potentially one of the micro-organisms that could use hydrogen during cecal fermentation [41]. 

Clearly, *Campylobacter* represent a serious threat to public health, but, aside from marginally effective hygienic and biosecurity measures, there are few practical interventions for controlling the colonization of food-producing animals with these pathogens [42,43]. From a public health perspective, new strategies are needed to reduce the incidence and concentration of these pathogens both on the farm and during processing. *Campylobacter* may not be competitive in a rumen environment, but the fact that *C. jejuni* does survive better in fecal mixed cultures suggests that the lower gastrointestinal tract ecology of *Campylobacter* colonization in both swine and ruminants may be a primary concern for transmission. In cattle, it has been established in several studies that *Campylobacter* are more likely to colonize the lower gastrointestinal tract [25]. In swine, Rath et al. [44] demonstrated with weaned pigs either infected with either *C. coli* and/or *C. jejuni* that both *Campylobacter* spp. could colonize the jejunum as well as the cecum. This would suggest that control measures implemented for limiting *Campylobacter* in swine and ruminants may need to target delivery to the lower gastrointestinal tract of these food animals.

In conclusion, it appears that *C. jejuni* can survive better in bovine fecal contents compared to rumen contents. While the environmental conditions of the rumen are likely more hostile to *Campylobacter*, other factors may also contribute to this difference. Certainly, the availability of substrates and potentially more competitive indigenous microbiota could contribute to the hostile nature of the rumen environment. Both the composition of fecal material and the presence of less competitive micro-organisms including hydrogen utilizers may be more favorable to *Campylobacter*’s survival. The presence of fecal metabolites such as amino acids may represent a greater availability of preferred substrates for *Campylobacter* metabolism versus rumen metabolite profiles. In addition, the fecal microbial population may be a more supportive microbial consortium that can potentially serve as cross feeders with *Campylobacter* by generating end-product metabolites that *Campylobacter* can use as substrates. However, the current study can only suggest potential factors. To determine which of these factors are contributors to *Campylobacter* survival in bovine fecal material will require 16S rDNA microbiome analyses to determine the microbial populations present and determine if specific relationships exist among certain fecal micro-organisms and *Campylobacter* levels. Metabolomic analyses would help elucidate which metabolites are present that could serve as potential substrates for *Campylobacter* metabolism. Combining these approaches in future studies offers an opportunity to better understand the ecology of *Campylobacter* under these conditions and potentially lead to more targeted mitigation strategies for this pathogen.

## Figures and Tables

**Table 1:** Comparisons of numbers of total culturable anaerobes, pH and ammonia concentrations after 48 h incubation of *Campylobacter jejuni* with mixed populations of bovine rumen or fecal micro-organisms or their combinations.

	*Campylobacter jejuni* Mean Specific Growth Rate (h^−1^)	Total Culturable Anaerobes (log_10_ CFUs/mL)	pH	Ammonia (µmol/mL)
Treatments	during initial 6 h incubation	After 48 h incubation		
*Campylobacter jejuni* only	0.222	NA ^†^	6.42 ^a^	1.25
2X Rumen fluid	0.145	7.13	6.18 ^b^	1.90
2X Feces	0.181	7.17	6.16 ^b^	1.75
1X Rumen fluid/1X feces	0.178	7.15	6.17 ^b^	1.83
Treatment effect	*p* = 0.1860	*p* = 0.7122	*p* = 0.0474	*p* = 0.1983
Standard error of the mean	0.022	0.037	0.062	0.211

^†^ NA; not applicable. ^a, b^ Means within columns with unlike superscripts differ at *p* < 0.05 based on an LSD multiple comparison of means.

**Table 2:** Main effects of initial gas phase, 100% carbon dioxide or a 50:50 carbon dioxide/hydrogen mix, and mechanistically different anti-methanogenic compounds, administered alone or together, on microbial and fermentation parameters during incubation of mixed fecal micro-organisms under a carbon dioxide alone or mixed with hydrogen and inoculated with *Campylobacter jejuni* to achieve 4.6 ± 0.1 log_10_ colony-forming units (CFUs)/mL incubation fluid.

	Change in *Campylobacter jejuni* (log_10_ CFUs/mL)		Gas Produced (mL)	Hydrogen Accumulation (µmol/mL)	Methane Accumulation (µmol/mL)	Nitrate Metabolized ^†^ (µmol/mL)
Parameter	After 6 h	After 24 h	After 24 h	After 24 h	After 24 h	After 24 h
Initial gas phase						
100% carbon dioxide	−0.12	−0.90	3.81 ^a^	0.52 ^b^	0.51 ^a^	2.06
50:50 Hydrogen/carbon dioxide	−0.07	−0.80	2.11 ^b^	32.07 ^a^	0.04 ^b^	1.99
*p* value	0.4617	0.2123	0.0296	<0.0001	0.0139	0.9233
SEM	0.050	0.055	0.517	0.854	0.123	0.523
Anti-methanogen treatment						
No treatment	−0.08	−0.72	3.75 ^x^	17.26	0.13	ND
0.05 mM 2-Bromosulfonate	−0.09	−0.80	4.87 ^x^	18.29	0.21	ND
5 mM sodium nitrate	−0.08	−0.99	1.50 ^y^	15.42	0.32	1.81
Combined	−0.13	−0.88	1.72 ^y^	14.20	0.43	2.24
*p* value	0.9628	0.0613	0.0011	0.9773	0.7465	0.5678
SEM	0.075	0.070	0.579	7.13	0.204	0.514

^†^ Comparisons of amounts of nitrate metabolized were made between cultures treated with nitrate alone or in combination with 2-bromosulfonate. ND; not done. ^a, b^ Means within columns with different superscripts differ at *p* < 0.05 based on an LSD multiple comparison of means. ^x, y^ Means within columns with different superscripts differ at *p* < 0.05 based on an LSD multiple comparison of means.

**Table 3:** Main effects of mechanistically different anti-methanogenic compounds, administered alone or together, on microbial and fermentation parameters during incubation of mixed fecal micro-organisms under a 100% carbon dioxide atmosphere and inoculated with *Campylobacter jejuni* to achieve 4.6 ± 0.1 log_10_ colony-forming units (CFUs)/mL incubation fluid.

	Change in *Campylobacter jejuni* (log_10_ CFUs/mL)		Gas Produced (mL)	Hydrogen Accumulation (µmol/mL)	Methane Accumulation (µmol/mL)	Ammonia Accumulation (µmol/mL)
Parameter	After 6 h	After 24 h	After 24 h	After 24 h	After 24 h	After 24 h
Anti-methanogen treatment						
No Treatment	−0.16	NA^†^	18.67	0.25	Undectable	0.96
0.05 mM 2-Bromosulfonate	−0.08	NA	17.67	0.15	Undectable	0.04
0.001 mM Monensin	−0.02	NA	19.00	0.54	Undectable	0.11
Combined	−0.14	NA	18.00	0.40	Undectable	0.19
*p* value	0.6080	-	0.1189	0.2086	-	0.0856
SEM	0.080	-	0.373	0.130	-	0.242

^†^ NA, not available due to unavoidable interruption of facilities services.

## Data Availability

Data is privately held but can be made available upon reasonable request.
